# Are PISA 2022 Creativity-Related Self-Reports Comparable Across Education Systems? Measurement Invariance, Alignment, and Associations with Creative-Thinking Performance

**DOI:** 10.3390/bs16101758

**Published:** 2026-09-27

**Authors:** Georgios Sideridis, Mohammed Alghamdi

**Affiliations:** 1 BARD, Boston Children’s Hospital, Boston, MA 02115, USA; 2 Department of Pediatrics, Harvard Medical School, Boston, MA 02115, USA; 3 Department of Self-Development Skills, King Saud University, Riyadh 11451, Saudi Arabia; mhalghamdi@ksu.edu.sa

**Keywords:** PISA 2022, creativity, measurement invariance, alignment optimisation, cross-cultural comparability, self-report scales, creative thinking, large-scale assessment

## Abstract

PISA 2022 creativity-related self-reports are used to study differences among education systems, but their interpretation depends on measurement structure, scoring and estimator choice. We audit six scales using item-level responses from up to 73 systems, the OECD item-response scaling record and associations with creative-thinking performance. Because the rotated questionnaire leaves almost no complete records, the primary models read weighted pairwise-present moments; the parameter estimates are inferential, but the fit indices and dispersion-reduction R^2^ computed from them are descriptive summaries of that approximation rather than survey-robust tests, and we mark which conclusions are which. The continuous models show substantial unidimensional misfit for four published indices. Loading constraints produce small fit decrements under the retained structures, scalar constraints larger ones. Alignment for four constructs concerns a selected item subset and cannot establish comparability of the published scale. Re-estimating all six by full-information marginal maximum likelihood on the ordinal responses, which handles the planned missingness and the response format exactly, lowers every loading R^2^ and rearranges their ordering (ρ = 0.14), leaves the intercept ordering essentially intact (ρ = 0.94), and reproduces the system means at r = 0.77 to 0.97 while moving 18% to 53% of systems by more than five rank positions. Within systems, observed-score associations are small and heterogeneous. Construct-level summaries are reasonably robust to these choices; individual system positions are not.

## 1. Introduction

PISA 2022 introduced a creative-thinking assessment and, alongside it, a battery of student self-report scales covering creative self-efficacy, creativity and openness to intellect, imagination and adventurousness, openness to art and reflection, and the creative environments students report at home and at school. These scales can be used to describe system differences and to model cross-national relationships involving creativity-related self-reports. Such use presumes that the scales measure the same constructs on the same metric everywhere, which requires examining the existing item-level scaling evidence alongside alternative measurement structures.

Background-questionnaire scales in international assessments have received less psychometric scrutiny than the cognitive instruments they accompany (Rutkowski & Rutkowski, 2010). Self-reports of attitudes and self-beliefs are vulnerable to translation differences, reference-group effects and response styles. Previous studies of international assessments have found departures from scalar invariance (Davidov et al., 2014; Pokropek et al., 2019). With many groups, exact invariance tests can detect small departures, so statistical rejection must be distinguished from their practical consequences (Kim et al., 2017; Rutkowski & Svetina, 2014). The question is which comparisons are defensible under particular models and assumptions, rather than whether a single label establishes comparability for every purpose.

Creativity is conventionally defined by novelty together with usefulness (Runco & Jaeger, 2012), is understood to be at least partly domain-specific (Baer, 1998; Plucker et al., 2004), to vary in how it is conceived across cultures (Glăveanu, 2010; Lubart, 1999; Niu & Sternberg, 2006), and to take different forms at different levels of attainment (Kaufman & Beghetto, 2009; Barbot et al., 2015). A battery of global self-reports is a coarse instrument for a construct of that shape, which is part of what is at stake here. Answering the comparability question requires distinguishing three questions that the literature often runs together. First, do the items measure the same construct within each system: is the measurement model defensible at all? Second, can system means be compared under defensible loading and intercept or threshold constraints? Third, do the constructs relate to measured creative performance in the same way everywhere? The three are logically separate, and a scale can pass one while failing another. The third question matters for secondary analyses, but measurement invariance alone cannot answer it.

Three properties of this battery shape what we should expect. Its items differ sharply in referent: some ask about concrete, externally observable behaviour (“I enjoy creating art”) and others for a global self-evaluation (“I have a good imagination”), and the second kind requires a comparison against an implicit standard that may itself differ across systems, a mechanism documented as reference-group bias in cross-national self-report (Heine et al., 2002; He et al., 2017). Several items are idiomatic and hard to render equivalently. One item is reverse-worded, a format whose behaviour differs across cultures and reading levels, and general response style is itself a documented source of cross-cultural incomparability in these instruments (He & van de Vijver, 2015). These are possible mechanisms, assessed here indirectly through item content and scaling patterns; the analysis does not isolate their causal contributions.

A further consideration is specific to this battery: the self-report scales and the criterion are matched at different grain sizes. The creative-thinking assessment is domain-structured, while the self-reports are global. Even under adequate measurement, this mismatch may reduce associations (Karwowski, 2014; Silvia et al., 2012), and it matters for how a small coefficient should be read. Goecke et al. (2025) have separately questioned whether the item pools of several of these scales are unified enough to support the constructs they name; our results bear on that directly.

The conceptual basis is the PISA creative-thinking framework, which distinguishes creative-task confidence, openness-related dispositions and perceived environmental support from demonstrated creative-thinking performance (OECD, 2023). Self-efficacy concerns confidence across expressive, scientific and social tasks; the openness-related pools mix enjoyment, challenge seeking, imagination, adventure, artistic production and reflection. The environmental scales concern perceived support from different social referents. The OECD technical report provides international IRT calibration, reliability and country-by-language parameter evidence; this is prior psychometric evidence, but does not by itself establish the particular correlated-factor structures used here. Goecke et al. (2025) identify ambiguity, construct underrepresentation and item overlap in these pools. Thus improved multidimensional fit is consistent with separable content domains but cannot distinguish substantive facets from wording or method effects. Only one reverse-worded imagination item is available, so a separate wording factor cannot be identified from that item alone. The facet labels are working descriptions of item content, not independently validated replacement constructs.

We examine six scales through three complementary analyses. Strand A fits multi-group factor models and alignment models to the microdata. Strand B examines the OECD’s independently produced generalised partial-credit scaling record for the same items (Muraki, 1992). Strand C computes item-level criterion gradients from published compendia. These analyses use the same underlying student responses and are not statistically independent replications. Our contribution is a conditional, item-set-specific account of the evidence and its limitations, rather than a certification that particular published indices can or cannot be used for every purpose.

## 2. Materials and Methods

### 2.1. Data

We analysed the PISA 2022 public-use student questionnaire file: 613,744 students in 80 participating countries and economies, and all 44 items of the six creativity-related scales (ST334 creative self-efficacy, ST336 the family and peer environment, ST340 creativity and openness to intellect, ST341 openness to art and reflection, ST342 imagination and adventurousness, ST335 the school and class environment). Item content, the referent classification used below, and the OECD’s international item slopes are given in Supplementary Table S1.

The two omitted student scales are participation in creative activities at school and participation in creative activities outside school (Goecke et al., 2025; OECD, 2024b). They concern reported participation or exposure to activities, whereas this audit is restricted to self-beliefs, dispositions and perceived support. Combining participation-frequency measures with this set would broaden the substantive target and require separate evaluation of response formats, opportunity constraints and scoring assumptions. This is a scope decision, not a result-based claim that those scales are less valid or less comparable. No conclusions are drawn about the two activity scales.

The criterion is creative-thinking performance. Its ten plausible values are held in the creative-thinking cognitive file rather than the questionnaire file, as PV1CRTH_NC to PV10CRTH_NC (499,843 students in 63 systems). Weighted country means computed from them reproduce the OECD’s published creative-thinking means at r = 1.000, which we treat as a check on the data pipeline. The published compendia cover 64 systems: the additional one is Cyprus, which is absent from the public-use student file, so analyses using the microdata rest on 63 systems and those using the compendia on 64. Sampling variance is estimated from the 80 Fay-adjusted balanced-repeated-replication weights, named W_FSTURWT1 to W_FSTURWT80 without a leading zero.

The moment-preparation code requires at least 100 valid responses to each analysed item in a system and a computable pairwise covariance matrix. In the supplied results, every exclusion had an item count of zero and coincided with the OECD record of non-administration (OECD, 2024b). Seven systems administered none of the six scales; Israel additionally administered neither self-efficacy nor the family and peer scale, and Norway did not administer imagination. Thus these numerical filters did not exclude an otherwise administered scale from the primary sample. The realised counts are 72 or 73 systems per construct for measurement analyses and 62 or 63 for criterion analyses. A separate sensitivity analysis excludes systems with poor continuous configural fit.

### 2.2. What the Rotated Design Gives Each Student

PISA 2022 used a rotated, planned-missing background questionnaire (Adams et al., 2013; Graham et al., 2006). Students typically answered five items per scale, and four for the analysed imagination items (Table 1). Almost no student answered every item of the longer scales; the five-item openness-to-art scale was complete for 96.5% of responding students. Observed means therefore combine different item subsets. Spearman–Brown calculations using the average inter-item correlation give illustrative reliabilities of 0.71–0.86 at the median observed item count, compared with 0.79–0.92 for complete scales. These approximations assume exchangeable items and cannot fully represent a multidimensional scale with changing content mixtures. The observed-score coefficients in Section 3.6 are descriptive; they are not necessarily lower bounds on latent associations, because item mixture and correlated measurement error can alter coefficients in either direction.

For multidimensional scales, the content mixture also varies across students. In the two ten-item scales, the aligned facet contributes half of the answered items on average, but approximately one fifth to four fifths at the tenth and ninetieth percentiles. We require at least two valid responses for an observed person-level mean; 99.1–99.2% of students with any response meet this criterion. Measurement models use weighted pairwise-present moments computed from the available item pairs. This approach uses the available pairs but is not a full-information likelihood for the rotated ordinal response data. Supplementary Tables S10 and S11 document coverage.

### 2.3. Measurement Models

For each construct in each system, we computed a weighted pairwise-present covariance matrix and mean vector, with the final student weight rescaled within the system. The recorded diagnostics show no positive-definite repair of the 435 covariance matrices. The continuous-model fit calculations and original alignment weights use an effective group size defined by the harmonic mean of the item-pair co-administration counts. This is an approximation for the incomplete response data, not a survey-design effective sample size.

In each system we fitted a unidimensional model, a correlated-factor model and a bifactor model using content-based facets: expressive, scientific and social content for self-efficacy; creative enjoyment and intellectual challenge for openness to intellect; peer and family support; making and reflecting on art; imagination and adventurousness; and teacher support and task design. The original selection rule required positive degrees of freedom and no boundary residual-variance solution in at least 90% of systems, then selected the most parsimonious eligible structure with median CFI ≥ 0.95. If none reached 0.95, it selected the best-fitting eligible structure. This was an exploratory selection rule, not evidence of adequate fit in every system. The audit of Supplementary Tables S5 and S6 found zero flagged Heywood cases in all six retained structures, so the 10% allowance did not admit any flagged system into the retained analyses; requiring zero such cases would retain the same models. Systems with poor fit were retained for transparent sensitivity assessment, not treated as having demonstrated configural equivalence. No conclusion about universal model adequacy follows from a median fit statistic.

The primary continuous-variable analyses minimise a normal-theory maximum-likelihood discrepancy using the weighted pairwise-present covariance matrices and mean vectors. Unweighted least squares is used in the separate polychoric-correlation sensitivity calculations. The primary fit statistics are not survey-robust likelihood tests for the incomplete ordinal raw data. We report CFI, RMSEA and SRMR as descriptive model diagnostics; RMSEA is interpreted cautiously with small degrees of freedom (Kenny et al., 2015). Total omega is computed from the retained loading, factor-covariance and residual-variance matrices. High reliability does not establish unidimensionality, invariance or content validity.

Two consequences of that approximation govern everything below, and separating them is more useful than a general caveat. The pairwise-present moment matrices are consistent for the population moments under the rotation’s planned missingness, so the quantities estimated from them—loadings, intercepts, factor covariances, the alignment transformation and the system means that follow from it—are estimates in the ordinary sense, and the bootstrap intervals we report for them are inferential with respect to the sampling of systems. What is not inferential is anything that depends on a likelihood. A pairwise-present moment matrix has no single sample size; the effective group size entering the fit calculations is a harmonic mean of item-pair co-administration counts rather than a survey-design quantity; and the χ^2^, CFI, RMSEA and SRMR computed from it are therefore descriptive summaries of discrepancy, not test statistics with known reference distributions. We use them to compare structures within a construct and to order constructs, never to accept or reject a model, and no *p*-value is attached to any of them. The dispersion-reduction R^2^ of Section 2.4 is descriptive in a second and stronger sense: it is an index of a particular implementation, its value depends on how many parameters the alignment must reconcile, and Section 3.3 shows how far it moves when the estimator changes.

Because that limitation cannot be argued away, we tested it. All six constructs were re-estimated under an estimator that makes neither approximation. In every system we fitted a unidimensional graded response model to the raw ordinal responses by marginal maximum likelihood with 25-point Gauss–Hermite quadrature, weighted by the final student weight. Full-information likelihood uses each student’s observed items only, so the planned missingness enters the likelihood directly rather than through pairwise moments, and the graded response model treats the four categories as ordered rather than as interval-scaled. The resulting loadings and category intercepts were then aligned with the same component-loss function, the same group-size pair weights and the same identification constraints as the continuous analyses, so that the two sets of results differ in the estimator and in nothing else. The item sets, economies and weights are those of the headline alignment, so each construct’s two results describe the same students answering the same items. The implementation, a check of its analytic gradient against numerical differentiation, and a recovery study on simulated data with planned missingness are given in Supplementary Tables S28–S30 and the scripts that accompany them.

### 2.4. Invariance and Alignment

For the retained continuous-variable structures, we fitted configural, metric and scalar models (Meredith, 1993; Millsap, 2011; Vandenberg & Lance, 2000). A CFI decrement of 0.010 is used as a descriptive reference, not a universal decision boundary (Chen, 2007; Cheung & Rensvold, 2002; Marsh et al., 2004). A small metric decrement is interpretable only relative to the adequacy of the configural model. Metric invariance establishes a common loading metric under that model; it does not establish equality, substantive validity or transportability of regression coefficients. Our custom alignment procedure minimises a smoothed component-loss function over loading and intercept differences, with group-size-based pair weights (Asparouhov & Muthén, 2014; Marsh et al., 2018; Muthén & Asparouhov, 2014). Its reported R^2^ statistics quantify the reduction in unweighted between-system item-parameter dispersion after transformation, clipped to [0, 1] in the implementation. They are descriptive indices of this implementation, not proportions of variance proven to arise from genuine construct differences. Intervals resample systems in 500 bootstrap replications and therefore do not propagate all student-sampling, item-selection or model-selection uncertainty.

The custom procedure aligns each eligible factor separately. Throughout the paper, a content facet of a construct and the item subset that carries it are the same thing under two names: the facet names describe the content, and “item subset” is used wherever the point at issue is that the result concerns part of a published scale rather than the whole of it. For four correlated-factor scales, the headline result uses a selected item subset: five expressive self-efficacy items, five creative-enjoyment items, three peer-support items, or three art-making items. School environment and the six analysed imagination items are aligned as single factors. Supplementary Table S14 reports the other eligible facets. The two-item scientific and art-reflection facets were not separately aligned under this implementation; this is a limitation of the adopted facet-level procedure, not a claim that such facets cannot be identified within a larger correlated-factor model. Excluding art-reflection items also excludes all of the openness-to-art items for which the OECD used national parameters. Consequently, the art-making result cannot establish comparability of openness to art and reflection as a complete construct. The same restriction applies to all selected-facet results. Facet order is an analysis convention, not independent evidence that the chosen facet is preferable.

### 2.5. The Withdrawn Classification Scheme

Earlier versions of this paper carried STRONG, MODERATE, WEAK and INSUFFICIENT labels for each construct, with cut-offs at configural CFI 0.90 and 0.95, metric ΔCFI 0.010 and intercept R^2^ 0.50 and 0.75. Those labels were developed after inspecting the results. They were not derived from a criterion, validated against an external standard, or specified in advance, and the sensitivity analyses reported below show that at least one of them turns on the estimator rather than on the construct. We have therefore removed them rather than relabelled them. Section 3.7 instead reports the evidence for each construct—unidimensional fit of the published items, the aligned item subset and its diagnostics, the OECD scaling record, the ordinal comparison and the criterion heterogeneity—with no category and no threshold applied to any column. Readers who want a summary judgement must form it from the evidence and state the cut-offs they used; nothing in this paper supplies them.

### 2.6. The Three Strands and Their Analyses

Strand A is the multi-group modelling and alignment of Section 2.3 and Section 2.4, estimated by us from the microdata. Strand B analyses the OECD’s published scaling record for these exact items (OECD, 2024b, Chapter 19 annexes): which item-by-group combinations required nationally estimated parameters, in which country-by-language groups, which scales were suppressed and where, and the reliability of each scale in each of the 92 to 94 groups. Strand C computes, from the creative-thinking compendia (OECD, 2024a), the criterion gradient of each item in each of the 64 systems that administered the assessment (63 for the two scales Israel did not administer): the slope of the four published category means on the category codes, in score points per response-category step. Both strands rest on the same underlying student responses as Strand A, so where they agree the agreement is between analyses with different failure modes, not between statistically independent samples.

### 2.7. Associations with Creative-Thinking Performance

Student-level analyses estimate within-system standardised associations using the mean of each student’s valid responses, standardised with the final student weight. These are observed-score analyses, not latent regressions. Avoiding the released unidimensional indices does not remove measurement error or make changing item mixtures comparable across students. The ten creative-thinking plausible values are combined using Rubin’s (1987) rules, with sampling variance from the 80 replicate weights. These procedures address the specified sampling and plausible-value uncertainty, but do not propagate uncertainty in the self-report measurement model.

The joint model includes all six observed-score predictors for 380,224 students in 62 systems with a score on every scale. A supplementary sensitivity calculation adjusts predictor correlations using full-scale omega, computed by the study’s own code rather than by a packaged routine; Section 2.8 gives the software and versions(Mplus 9). Because omega refers to a retained model and students answer different item subsets, that adjustment is approximate and is not equivalent to latent-variable estimation. It cannot separate collinearity, measurement error and changing content mixtures. Questionnaire rotation permits the joint observed-score analysis, but does not remove these interpretive limitations.

System-specific coefficients are synthesised explicitly rather than averaged. We report a DerSimonian–Laird random-effects estimate with its 95% interval, the between-system standard deviation τ, and I^2^. Supplementary Table S9 adds the standard error of each estimate, a Paule–Mandel estimate as a check, τ^2^ with Cochran’s Q, a 95% prediction interval, a fixed-effect estimate, and a nonparametric bootstrap resampling economies, which is the resampling unit an unweighted mean implies. Heterogeneity is summarised by τ, the standard deviation and the interquartile range rather than by a coefficient of variation, which is uninterpretable where the mean approaches zero—as it does for the school-environment scale, whose system-specific coefficients change sign. Because effect magnitude and heterogeneity are more informative than significance alone, every coefficient is reported with an interval and with the share of criterion variance it accounts for, and the counts of system-specific coefficients reaching significance are given after Holm and Benjamini–Hochberg adjustment across the 62 or 63 tests as well as before.

At system level, we correlate mean creative-thinking performance with custom alignment means and with unadjusted item means. We report Fisher-z intervals and Williams tests for dependent correlations (Williams, 1959; Steiger, 1980), with Benjamini–Hochberg adjustment across six tests. For selected item subsets, both whole-scale and item-matched unadjusted means are used. These are descriptive sensitivity comparisons, not validation of latent system means. Additional adjustment includes system mean mathematics performance and economic, social and cultural status. Language, region, shared translations and dependence among culturally related systems are not modelled. The intervals and tests also condition on estimated alignment means and treat systems as independent; they may understate uncertainty and cannot support causal or population-wide generalisation.

### 2.8. Software, Validation and Reproducibility

The original pipeline used Python 3.11 with NumPy, SciPy, pandas and pyreadstat. The revision audit used Python 3.12.14 with NumPy 2.3.5, SciPy 1.15.3 and pandas 3.0.1, together with Mplus 9; its full environment record, all new inputs and all outputs are retained in the analysis archive. The supplied code implements polychoric calculations, moment-based factor models, a custom alignment optimiser and replicate-weight analyses. We distinguish verification of code execution, internal numerical checks and comparison with an independent implementation; none is a substitute for evaluating the measurement model or the assumptions governing missing responses.

The original self-test recovers simulated group means after centering and scale adjustment and rejects a unidimensional model for two-facet data. Its reported zero average bias is not an independent unbiasedness check: both true and estimated group means are centered. The configural separability check verifies agreement between the joint configural objective and the sum of group-specific objectives to within 5 × 10^−8^. The expanded simulation in Section 3.4 assesses recovery under a specified data-generating model. It is an internal sensitivity analysis and does not independently validate the alignment implementation. The Mplus comparisons in Section 3.3 serve a separate purpose.

The earlier Mplus benchmark combined a change of estimator with changes in item selection and factor structure. The revision separates full-scale categorical findings from item-matched categorical and continuous alignment comparisons. Matching the items removes the item-selection difference, but does not automatically equate the first-stage estimates from a standalone facet model and a facet extracted from a correlated-factor model. The software audit therefore also reconstructs a common configural solution from Mplus estimates and evaluates alignment on those parameters. Identification, smoothing, group weighting, output precision and any Mplus warnings are recorded with the comparison. Agreement is evaluated quantitatively rather than assumed from successful termination; results from models with unresolved warnings remain provisional.

For the matched Mplus comparisons, each estimator reads the same selected-item data, after removing rows with no observed facet item and renormalizing the supplied student weights within systems. Both use MLR; the categorical specification uses 15 integration points. Shared fixed-reference comparisons are used when a free-alignment warning motivates that sensitivity check; each reported pair uses the same alignment identification, and the raw outputs record the selected reference value and any remaining warning. Correlations compare means after centering and rescaling to a geometric mean factor standard deviation of one. Separately, categorical WLSMV models with DELTA parameterisation evaluate the retained full factor patterns. Explicitly identified configural models allow group-specific loadings and thresholds, with factor means zero, factor variances one and ordinal scale factors one in each group. The same retained patterns are also fitted separately in each system to check local fit and admissibility. Scalar comparisons and any unavailable difference tests are reported separately. These weighted checks hold the relevant item sets constant but do not incorporate the full replication-weight survey design.

We also report three errors found and corrected in our own code during development, because all three would have produced plausible-looking results. The multi-group routine fixed the factor covariance matrix to the identity, fitting orthogonal factors where the model specified correlated ones, which depressed configural CFI by 0.13 to 0.24 for the four correlated-factor constructs. The fit-index routine divided by degrees of freedom without checking that they were positive, so a just-identified bifactor model returned a CFI near 1.00 as an arithmetic artefact. The reliability routine summed squared item communalities where McDonald’s omega requires the squared sum of loadings carried through the factor covariance matrix. All three are corrected; the separability check guards the first, and every number here has been recomputed.

## 3. Results

### 3.1. Measurement Structure and System-Specific Fit

The continuous unidimensional model fits the school scale relatively well (median CFI 0.976; no system below 0.90). Median CFI is 0.944 for openness to art and 0.953 for the six analysed imagination items, with six imagination systems below 0.90. Misfit is more widespread for self-efficacy (0.888; 41/72 systems below 0.90), openness to intellect (0.849; 57/73) and the family/peer scale (0.788; 68/72). Including the reverse-worded imagination item gives median CFI of 0.846, with 63/72 systems below 0.90. These findings question the unidimensional interpretation of four released indices under this continuous-variable analysis. They do not constitute a categorical re-estimation of the OECD scaling model or a demonstration that the indices are unusable for every purpose.

The bifactor alternative has the best fit among the three specifications wherever it is defined (median CFI 0.962 to 0.998). None is retained. For openness to art it is just-identified, so no fit index exists for it; for self-efficacy, openness to intellect, the school environment and imagination it yields inadmissible solutions in 24% to 47% of economies; and for the family and peer scale, where it is both defined and admissible, the correlated-factor model already reaches a median CFI of 0.993, so the parsimony rule retains the simpler structure. Because no general-plus-specific structure is retained, we do not report the explained-common-variance and omega-hierarchical indices with which such models are judged (Reise, 2012; Rodriguez et al., 2016): they can be computed for the five constructs whose bifactor model is identified, and are given in Supplementary Table S5, but they describe a structure this study does not use. The retained structures are unidimensional for the school environment and imagination and correlated factors for the other four, with model-based omega between 0.78 and 0.94 (Table 2).

Openness to intellect remains particularly uncertain: the retained correlated-factor structure has median CFI 0.915 and pooled configural CFI 0.919. Twenty-one of 73 systems have CFI below 0.90. Self-efficacy has three such systems (Korea, Panama and El Salvador), and imagination has six (Austria, Denmark, Korea, the Netherlands, New Zealand and Thailand). None of the retained continuous structures has a recorded Heywood case, but numerical admissibility and substantive model adequacy are separate requirements. All comparisons for openness to intellect, and conclusions involving the poorly fitting systems for other scales, remain provisional. Per-system fit and sensitivity results are supplied rather than using acceptable median fit to imply adequacy everywhere.

Separate ordinal WLSMV fits of the same retained patterns were also completed for all 435 construct-by-system combinations (Supplementary Tables S24 and S25). Of these, 434 had no flagged admissibility warning. The separate self-efficacy fit in Panama produced a non-positive-definite factor covariance matrix, with expressive–scientific and social–scientific correlations of 1.005 and 1.037. The pooled configural run did not flag that problem, so its favourable overall CFI cannot establish admissibility in every separate system fit. Among the remaining fits, CFI ranged from 0.932 to 1.000; none was below 0.90, and six imagination fits were below 0.95. Openness-to-intellect CFI ranged from 0.966 to 0.997 under ordinal estimation, contrasting with its continuous-model shortfall. RMSEA exceeded 0.10 in 19 art/reflection fits, 15 school-environment fits, two imagination fits and one family/peer fit. These diagnostics require joint interpretation, particularly for the four-degree-of-freedom art/reflection model; favourable CFI alone is not a claim of uniformly adequate fit.

Excluding systems with continuous configural CFI below 0.90 changes the custom intercept R^2^ from 0.803 to 0.790 for expressive self-efficacy, from 0.699 to 0.687 for creative enjoyment, and from 0.402 to 0.433 for imagination. Correlations of the restricted-sample means with the original means exceed 0.999 in these comparisons. This sensitivity suggests that these particular point estimates are not driven by the excluded systems; it does not establish comparability for the excluded systems or remedy weak full-scale fit. See Supplementary Table S22.

Because the retained structures were selected in the same systems used to evaluate them, we quantified how far the selection itself is reproducible. The rule is mechanical, so it can be re-applied to a subset: splitting the systems at random into halves 5000 times and applying it to one half alone reproduces the full-sample structure in 100% of splits for five of the six constructs. The exception is imagination, where the unidimensional model is selected in 83.7% of half-samples and the correlated-factor model in the remaining 16.3%: its unidimensional median CFI of 0.953 [0.944, 0.960] straddles the 0.95 criterion, so which structure the rule returns depends on which systems are in the sample. This has not been flagged before and should be. Openness to intellect is selected unanimously, but for the opposite reason and with the opposite implication: its correlated-factor median CFI is 0.915 [0.908, 0.924], an interval lying wholly below 0.95, so in every split the rule falls through to its fallback clause and returns the best-fitting admissible structure rather than an adequate one. What makes that construct provisional is therefore not uncertainty about which structure was chosen—it is that no candidate structure is adequate, in any half of the data. Supplementary Table S31 gives the full specification curve: median CFI with bootstrap interval, share of systems below 0.90, and Heywood rate for all three candidate structures in all six constructs.

### 3.2. Invariance Evidence Depends on the Estimator and Structure

Under the retained continuous-variable structures, loading constraints produce CFI decrements of approximately 0.004–0.006 for five scales and 0.010 for imagination. Scalar constraints produce larger decrements, 0.015–0.078 (Table 2). Figure 1 shows the configural, metric and scalar fit of each construct side by side. This pattern suggests that loading equality is a less restrictive approximation than intercept equality in these particular models. It does not demonstrate estimator-independent metric invariance for five complete constructs. Openness to intellect has weak configural fit; some other systems also fit poorly, and alignment uses selected item subsets for four scales. A common measurement metric would permit a meaningful test of structural relations under a suitable model, but would not establish that those relations are equal or valid across systems.

Two categorical scalar attempts were unusable: creative self-efficacy had an inadmissible factor correlation of 1.007 between expressive and scientific self-efficacy in Panama (G56); openness to intellect did not converge. We do not interpret these estimation or admissibility problems as a statistical rejection or as support for invariance.

We additionally fitted categorical WLSMV models with the retained full factor patterns, rather than only the headline facets. Pooled configural runs without flagged warnings gave creative self-efficacy CFI 0.996; school environment CFI 0.990; family and peer environment CFI 0.997; openness to intellect CFI 0.988; openness to art and reflection CFI 0.998; imagination CFI 0.980. Configural RMSEA ranged from 0.038 to 0.089. Corresponding scalar CFI values were school environment 0.984 (decrement 0.006); family and peer environment 0.993 (decrement 0.004); openness to art and reflection 0.994 (decrement 0.004); imagination 0.945 (decrement 0.035). These ordinal decrements do not reproduce a uniform pattern of scalar misfit and qualify the continuous-model findings. Supplementary Table S23 gives model-specific fit and warnings. Configural models use explicit unit factor variances and ordinal scale factors within each group. Some automatic configural runs failed; for those cases, the reported scalar point fit is compared descriptively with the separately identified configural fit, without claiming an available corrected chi-square difference test. An unavailable or inadmissible solution does not count as evidence for invariance. These analyses use student weights but do not provide a complete complex-survey ordinal invariance analysis.

### 3.3. Approximate Comparability, and What the Benchmark Does and Does Not Establish

The dispersion-reduction intercept R^2^ values are 0.90 [0.87, 0.92] for art making, 0.88 [0.84, 0.91] for school environment, 0.80 [0.74, 0.85] for expressive self-efficacy, 0.79 [0.73, 0.85] for peer support, 0.70 [0.62, 0.77] for creative enjoyment and 0.40 [0.32, 0.48] for imagination (Table 3). Figure 2 plots these indices, and the corresponding values for the loadings, with their intervals. Each describes the specified transformation of the selected item parameters and none is the proportion of cross-national variation known to be a genuine creativity difference. Two of the six have intervals wide enough to reach well below their point estimates, and the constructs cannot be ranked against one another on these numbers alone because they rest on different item sets and different measurement structures. This is the quantity the classification scheme used as its principal input, and it is the quantity the ordinal re-estimation below moves the most.

The choice of item subset changes the substantive target, and by enough to matter. Art making excludes the two reflection items responsible for all 16 nationally estimated openness-to-art parameters in the OECD record. Expressive self-efficacy has intercept R^2^ 0.80 where the social subset has 0.88; creative enjoyment has 0.70 against 0.69 for intellectual challenge; peer support has 0.79 against 0.70 for family support. The headline figure for four of the six constructs is therefore a figure about three to five items, not about the published scale, and neither the high art-making value nor the peer-support value can be carried across to the scale the OECD scores and releases. Supplementary Table S14 identifies the items and reports every eligible subset, including those excluded and the limitations of aligning them separately.

Rank positions make the practical stake concrete. Moving from naive item means over the whole scale to aligned latent means changes the school-environment ordering little—no system shifts more than three places—but 42 of 72 systems shift more than five places for the family and peer scale, the largest by 40; 30 of 73 for openness to intellect, and 23 of 72 for self-efficacy. Read as a statement about alignment, that comparison is misleading, and the correction is instructive. For a correlated-factor construct, a naive mean over the whole scale differs from the aligned mean in two ways at once. Recomputing the naive means over exactly the aligned items, the family and peer figure falls from 42 systems to 5 and its largest shift from 40 places to 10; openness to intellect falls from 30 to 10, self-efficacy from 23 to 2, and openness to art from 14 to 3, while the two unidimensional scales cannot be affected and stay at 0 and 5. Alignment does move systems, but most of the movement is a consequence of scoring half a scale rather than all of it.

The original full-scale categorical Mplus benchmark yielded correlations of approximately 0.97, 0.94, 0.90, 0.84, 0.69 and 0.59 with the custom means for school environment, self-efficacy, openness to intellect, openness to art, imagination and family/peer environment, respectively. These results remain documented as unmatched comparisons. For four scales, they combine differences in item selection, measurement structure, first-stage estimation and alignment implementation. They cannot isolate an ordinal-estimator effect. Correlations across only six scale-level summaries do not identify the cause of the disagreement.

The full-scale categorical outputs flag approximately 33–60% of item-by-group parameters and 39–62% of thresholds as non-invariant. These are significance-based flags under those particular models, not directly comparable to the custom R^2^ dispersion measures or the OECD decisions to use national parameters. Large samples may make small differences detectable, but this does not establish that the flags are inflated or that their practical effects are negligible. We withdraw the earlier claims that continuous estimates are lower bounds and categorical flags are upper bounds on consequential non-invariance. The matched analyses are required to evaluate sensitivity while holding the item set constant.

The new item-matched Mplus comparison gives the following correlations between categorical and continuous system means: school environment 0.916; expressive self-efficacy 0.963; creative enjoyment 0.847; art making 0.263; imagination 0.658; peer support 0.703 (Supplementary Table S19). Both estimates in each pair concern the same items. Estimation warnings remain for art making, and those comparisons are provisional. These comparisons remove the full-scale versus selected-facet difference, but do not establish that any set of means is unbiased or that the corresponding full construct is comparable.

A second sensitivity analysis addresses the estimator itself rather than the item set, and it separates the two indices sharply. Fitting a graded response model in every system by full-information marginal maximum likelihood, which handles the planned missingness and the four ordered categories exactly, and aligning it identically, lowers the loading R^2^ for all six constructs by between 0.09 and 0.33, and rearranges them: the ordering of the six on that index under the two estimators correlates at ρ = 0.14, which is to say it does not survive. The intercept R^2^, computed on the item-mean intercept so that both estimators carry one parameter per item, behaves quite differently. It changes by −0.04 on average, falls for five of the six and rises for the other, and the construct ordering is essentially preserved (ρ = 0.94): openness to art and school environment remain first and second, imagination last, and the only change is that openness to intellect and the family and peer scale swap adjacent places. Computed over all three category intercepts of each item, the values are lower throughout, but that reading is not like-for-like, since three parameters per item must be reconciled through a single group mean; Table 3 and Supplementary Table S28 report both.

Two consequences follow, and the first is unwelcome. Creative self-efficacy’s intercept R^2^ of 0.80, which was the basis for its former higher classification, becomes 0.73 under the ordinal estimator—on the other side of the 0.75 line the classification used. A summary that changes side when the estimator changes is not a property of the construct, and this is one of the two reasons the classifications have been withdrawn (Section 2.5). The second is that a reader should treat the loading index and the intercept index quite differently. The intercept index is telling us something about the constructs that two very different estimators agree on; the loading index is telling us at least as much about the estimator as about the construct, and the paper does not rest any conclusion on the ordering of the six constructs by loading R^2^.

The system means are the more robust quantity, and the contrast with individual positions is the useful result. Across the six constructs the two estimators agree on the aligned means at r = 0.77 to 0.97 (median 0.92), the weakest agreement being the family and peer scale and the strongest creative self-efficacy. Individual positions are another matter: between 18% and 53% of systems move more than five rank places depending on the construct, and the largest single displacement is 47 places. This is the same asymmetry the simulation in Section 3.4 produces within its own design and the item-matched Mplus comparison shows for the same six item sets, now with the planned missingness and the response format handled exactly: broad construct-level statements survive a change of estimator, and the position of any particular system does not. Supplementary Table S29 reports the per-system means under both estimators, so that a reader interested in one system can see how far it moves.

The independent comparison additionally matches Mplus’s internal parameter scaling. Mplus reports its alignment loss in a product-variance metric (Asparouhov & Muthén, 2023). We reconstruct common configural parameters from its printed optimisation-metric loadings, intercepts, means and variances, and apply the same component-loss routine with the corresponding fixed or free mean-origin condition, product-one variances, group-size weights and smoothing value 0.01. The results are r > 0.99999 for all six facets, mean absolute differences of 0.0001–0.0005, and a largest absolute difference of 0.0009; 180 of 180 independent starts converged (Supplementary Table S27). Three-decimal printing limits exact agreement. This is a check of the routine under matched inputs and settings; it does not establish unbiased means or validate every original custom result. The earlier raw-reporting-metric comparison and the original centered-mean routine are retained in Table S26, making the consequences of parameter scaling and identification explicit.

### 3.4. Simulation Evidence Under Specified Assumptions

The simulation answers one question and no other: if system means differ by a known amount and a known share of item parameters are non-invariant, how well does an alignment of the kind used here recover the ordering of those means? It is not a model of PISA, and it cannot be read as one. It does not reproduce the actual system sample sizes, the five- to ten-item scale lengths, the retained correlated-factor structures or the questionnaire rotation, and its first-stage extraction is the leading eigenvector of the correlation matrix rather than the CFA routine used on the data. Its conditions cross four proportions of perturbed item-by-group cells (0%, 25%, 40%, 60%) with four standard deviations of true system means (0.15, 0.20, 0.24, 0.40), at 300 replications of 73 groups of 1500 observations on six four-category items, with random item masking to a mean of 4.9 observed items.

Within that design (Table 4), recovering the ordering and recovering individual positions come apart. At a true spread of 0.20 SD, the mean correlation between estimated and true system means falls from 0.984 with no planted non-invariance to 0.860 at 60% perturbed cells, while the mean share of systems displaced by more than five ranks rises from 18.4% to 52.4%; Monte Carlo standard errors for the correlation range from 0.0001 to 0.0034 across the 16 conditions. Two statistics reported in earlier versions are withdrawn rather than updated: average bias is zero by construction, because both sets of means are centred, and the reported RMSE used a scale multiplier chosen with reference to the true means, which makes it an optimistically calibrated descriptive error rather than a recovery error on a prespecified metric. Neither licenses any claim about bias in PISA, and the displacement percentages describe this simulation rather than estimated error rates for real system rankings.

### 3.5. The Item-Level Record and Where Non-Invariance Concentrates

The OECD’s own scaling record separates the battery sharply. For self-efficacy, the family and peer scale and the school environment, every item was scaled with the international parameters in every one of the 92 to 94 country-by-language groups. For the other three, 43 item-by-group combinations departed from the international calibration: 20 in openness to intellect (2.13% of its 940 item-by-group parameters, in 19 groups), 16 in openness to art (3.40% of 470, in 10 groups) and 7 in imagination (1.09% of 644, in 7 groups). The item tables list 42 rows rather than 43 because one row reports a single set of parameters for Mongolia’s Mongolian- and Kazakh-language groups together; we count groups.

The deviations are not scattered. Thirty-six of the 43 fall on four items: “I like school work that is challenging” (16 groups), “I reflect on movies I watch” (10), “I see beauty in everyday things” (6) and the reverse-worded “I have difficulty using my imagination” (4). These are, respectively, one of the two weakest-discriminating items in its scale, the two weakest in theirs, and an item whose international slope is indistinguishable from zero. These departures cluster among items with low international discrimination, although three comparably weak items in other scales deviated in no group. This descriptive concentration does not establish a general predictive rule.

The two aesthetic-idiom items deviated exclusively in Albanian-, Macedonian-, Russian-, Ukrainian-, Kazakh-, Arabic-, Malay- and Thai-speaking groups, and 11 of the challenge item’s 16 groups are Spanish-, Arabic- or Albanian-speaking, the remainder being French-, Macedonian-, English- and Ukrainian- or Russian-speaking. We did not examine the translations themselves, and language, region and culture are confounded in these data, so the correct statement is that the pattern is consistent with translation-related differences rather than that translations diverge. It supports the expectation about item referent only weakly and only in places: cleanly within openness to art, where exactly the two abstract items deviate, and not at all within openness to intellect, where three of four deviating items have external referents. Across the 42 rows of the item tables, abstract items attracted 22, a rate of 1.22 per abstract item against 0.77 per concrete item—in the predicted direction but resting on nine items. These descriptive patterns motivate tests of wording and translation effects, but do not establish their relative contributions.

The record also carries the reliability evidence. Imagination’s alpha, as the OECD computes it on the published seven-item scale, averages 0.635 and falls below 0.70 in 87 of 92 groups and as low as 0.52, and the OECD suppressed its scale scores in 14 countries. That figure is not comparable with the omega we report in Table 2, which is a different coefficient computed under a different model on the six analysed items; the two agree in placing imagination last, and that agreement is what we rely on.

### 3.6. Relationships with Creative-Thinking Performance

The pooled within-system observed-score associations are small and positive (Table 5): imagination 0.217 [0.206, 0.228], openness to intellect 0.168, family/peer environment 0.144, openness to art 0.128, self-efficacy 0.126 and school environment 0.052 [0.031, 0.072]. These are associations between the available-item means and creative-thinking performance. Squaring a pooled coefficient gives a descriptive squared summary correlation; it is not generally the average within-system variance explained. The random-effects results do not imply that every system-specific coefficient is positive or that these are error-free latent relations.

The pooled figures conceal heterogeneity that is itself the finding. Between-system standard deviations τ run from 0.036 to 0.081 and I^2^ from 77% to 94%, so the system-specific coefficients differ far more than sampling error allows. The school environment is the extreme case: its coefficients have the largest spread in the battery (SD 0.076, interquartile range −0.01 to +0.10) and are zero or negative in 17 of 63 systems, significantly so in 6 before and 2 after Holm adjustment. A single pooled coefficient obscures this substantial between-system variation.

Because the six dispositions are correlated between 0.17 and 0.54, a bivariate coefficient is not a unique contribution. Entering all six jointly, on the 380,224 students with a score on every scale, the joint model accounts for 7.0% of criterion variance against 18.0% for the six bivariate associations summed—both computed within each system and then averaged, so that the two are like for like. The ratio averages 0.42 across systems (bootstrap 95% CI 0.39 to 0.45; median 0.40) and is below one half in 49 of the 62 systems.

The shrinkage is uneven (Table 5). Imagination retains most of its association (0.159), the family and peer scale about half (0.078) and openness to intellect two-fifths (0.069), all three remaining positive in nearly every system. Self-efficacy loses most of its association (0.020) and no longer has a consistent sign. Openness to art falls to −0.002 [−0.017, +0.013], a coefficient effectively null rather than reversed. The school environment falls to −0.043 [−0.059, −0.027], negative in 50 of 62 systems and significantly so in 18 after Holm adjustment.

The negative school-environment partial coefficient is conditional on the other observed scores and is not a causal effect of creative schooling. Predictor overlap, measurement error, changing facet mixtures, omitted variables and suppression may all contribute to the change from bivariate to joint coefficients. The reliability-adjusted sensitivity analysis does not identify which mechanism is responsible and is not an approximation that validates a latent regression. We therefore interpret the joint model as a description of conditional observed-score associations, not as a test of the unique causal or latent contribution of each disposition.

Between systems, the original custom alignment means correlate with performance at −0.56 for art making, −0.48 for school environment, −0.32 for creative enjoyment and −0.19 for expressive self-efficacy, compared with +0.47 for imagination and +0.26 for peer support. Figure 3 sets these between-system correlations against the pooled within-system coefficients. These descriptive contrasts resemble aspects of the attitude–achievement patterns reported for academic self-concept (Marsh & Hau, 2003; Van de Gaer et al., 2012), but they involve different estimands from the within-system observed-score coefficients. Intervals are wide with 62 or 63 systems. Adjusting for mean mathematics performance and economic, social and cultural status changes the sign for self-efficacy and creative enjoyment and reduces the peer-support coefficient towards zero. This sensitivity, omitted system-level dependencies and uncertainty in the alignment means preclude a general ecological or causal interpretation.

Whether the choice between naive and aligned system means changes these conclusions has a two-part answer. Against naive means over the whole scale, the two scorings give significantly different criterion correlations for four of six constructs raw and four after Benjamini–Hochberg adjustment, though only two survive the stricter Holm correction; the correlation moves from −0.34 to −0.19 for self-efficacy and from −0.44 to −0.32 for openness to intellect. This compares two constructed scores, not a direct comparison with the released OECD index. It is not a measure of what alignment does, because for four constructs the aligned mean is one facet’s and the naive mean the whole scale’s. With the item set held constant, the difference is significant for five of six but at most 0.07 in size, and it changes direction for three of the four correlated-factor constructs. The larger effect comes from which items are scored: for self-efficacy the facet-versus-scale change is five times the alignment change, and for openness to intellect nearly three times. Supplementary Table S15 gives both comparisons.

A further sensitivity comparison holds the participating systems and facet items constant while using the new Mplus means. The performance correlations for continuous/categorical estimation are school environment: −0.477/−0.353; expressive self-efficacy: −0.163/−0.075; creative enjoyment: −0.296/−0.354; art making: −0.570/−0.481; imagination: 0.598/0.150; peer support: 0.300/0.300 (Supplementary Table S21). The largest absolute change is for imagination (0.448), making its between-system interpretation particularly sensitive to estimation. These are descriptive correlations conditional on the estimated means; warnings in Table S19 also apply here. Similar criterion correlations need not imply close agreement of the system means themselves, and differences do not by themselves establish validity failure.

### 3.7. The Evidence Profile, and Criterion Heterogeneity

Table 6 sets the evidence for each construct side by side without classifying any of it. Read across a row, the recurring pattern is that no construct is favourable on every column. School environment has the best-fitting published item set—median CFI 0.976 with no system below 0.90—and the largest criterion heterogeneity in the battery. Openness to art has the highest intercept R^2^ under both estimators, but that figure belongs to three making items and excludes the two reflection items responsible for all sixteen of its nationally estimated parameters, and its categorical comparison carries an unresolved warning. Self-efficacy is third on that index, and its value crosses the boundary the former classification used as soon as the estimator changes (Section 3.3). Openness to intellect has no adequate structure in any half of the data, though its intercept R^2^ is the one that does not fall under the ordinal estimator. Imagination has the lowest intercept R^2^ in the battery and a structure selection that does not reproduce. The family and peer scale has the weakest agreement between estimators on its system means. Every construct is favourable somewhere and unfavourable somewhere else, which is why a single label was never going to carry the information, and why the paper no longer offers one.

School environment combines relatively favourable continuous measurement diagnostics with substantial heterogeneity in its observed relation to performance. The compendium-based item gradients are zero or negative in 13 of 64 systems; student-level coefficients are zero or negative in 17 of 63; and its joint observed-score coefficient is negative in most systems. These overlapping analyses describe a pattern requiring explanation. They do not demonstrate that the scale lacks validity in particular systems or that measurement comparability and validity necessarily diverge: item mixtures, measurement error, population composition, omitted variables and genuine moderation remain competing explanations.

## 4. Discussion

### 4.1. How Far the Three Questions Can Be Answered

The primary continuous analysis identifies substantial unidimensional misfit for four published indices. Content-based alternatives improve fit, but they were selected exploratorily and do not fit all systems well. The revised analysis distinguishes this structural question from loading and threshold invariance and from substantive relations with performance. The results complement the content-validity concerns of Goecke et al. (2025), but cannot determine from fit alone whether the misfit reflects substantive multidimensionality, item overlap, wording effects or imprecise construct definitions. Nor do they imply that the OECD undertook no validation: the OECD scaling record is an important source of evidence in this audit.

For mean comparisons, the continuous scalar models fit worse than their metric counterparts. Approximate alignment results depend on item selection, estimation and identification, and favourable values for a selected item subset do not generalise to a full scale. The expanded simulation is informative about its specified conditions but does not establish unbiased recovery for actual systems. We therefore replace definitive permissions and prohibitions with provisional conclusions about the strength and limits of the evidence. An aggregate comparison requires justification for the particular item set, population and estimand, with sensitivity to alternative measurement models and estimators; no summary of the kind this paper previously offered supplies any of them, which is why it no longer offers one. The retained full-pattern ordinal models also yield small scalar CFI changes for some scales, so the continuous scalar pattern cannot be generalised across estimators.

On the third question—how the dispositions relate to creative performance—the findings both reproduce and complicate the existing picture. The pooled within-system associations are positive and small, and entering the six jointly leaves well under half of what they explain separately. The magnitudes are smaller than the creative-self-beliefs literature would suggest (Karwowski, 2014; Silvia et al., 2012), and the gap is instructive: those literatures relate self-beliefs to divergent-thinking or self-reported-activity criteria in the same domain, whereas PISA relates global self-beliefs to a domain-structured performance assessment. The grain-size mismatch is a design feature of the battery rather than a finding about creativity, and changing item mixtures and measurement error further complicate the comparison. Between systems most associations are negative, reproducing the attitude–achievement pattern only partially, since two are positive and adjustment for development level reverses two more.

### 4.2. Why Comparability Varies

The OECD item record suggests that a small number of items merit closer investigation: the reverse-worded imagination item, two art-reflection items and the academic-challenge item. These patterns are consistent with content or wording differences, but translations were not examined and response-style or reference-group explanations were not directly tested. The appropriate follow-up is content validation, cognitive interviewing and translation review. Removing these items could also narrow construct coverage, so improved comparability cannot be assumed.

### 4.3. Measurement Comparability and Heterogeneous Criterion Relations

Measurement invariance and equality of structural relations are distinct hypotheses. Even an adequate invariant measurement model need not imply equal regression coefficients across populations. Conversely, heterogeneous observed-score coefficients do not establish validity non-comparability. The school-environment pattern motivates that hypothesis, but testing it requires latent and multilevel models that account for measurement error, item mixtures, population composition and potential moderation. We therefore describe criterion heterogeneity rather than asserting that the best-measured scale has the least valid criterion relation.

### 4.4. Implications

For secondary analysts, the recommendations are provisional and specific to the intended comparison. The school environment has relatively favourable continuous diagnostics, but categorical non-invariance still requires scrutiny. Selected self-efficacy and peer-support facets cannot substitute for full-scale evidence. Art-making results exclude the reflection items that show OECD parameter departures, and the categorical art-making model retains a singularity warning. For openness to intellect, weak continuous configural fit contrasts with more favourable ordinal configural CFI, but the ordinal scalar attempt failed to converge. Imagination is sensitive to item inclusion, has a lower custom intercept-dispersion index, and shows appreciable estimator sensitivity in its matched Mplus means and between-system performance correlation. These patterns identify priorities for sensitivity analysis, not universal permission or prohibition to compare system means. Researchers should report the exact items, factor structure, estimator, identification, missing-data treatment and uncertainty underlying each comparison.

Association analyses should present system-specific estimates and the distribution of heterogeneity alongside pooled summaries. Bivariate and joint models answer different questions; a smaller joint coefficient does not show that a bivariate association was incorrectly estimated. Both should be identified as observed-score analyses when based on available-item means. Latent regressions with compatible item structures, and multilevel models of slope variation, are needed to distinguish measurement effects from substantive moderation. Between-system analyses should additionally assess language, region and dependence among related systems.

Self-report system differences and creative-thinking performance differences concern distinct constructs and levels of inference. Their juxtaposition should not suggest that a higher self-report score implies higher demonstrated creative performance. Cross-sectional observed-score associations provide no causal evidence for or against a classroom practice. Policy interpretation requires attention to measurement uncertainty, opportunity differences and the population to which a claim is intended to apply.

Four items account for most departures from international item parameters in the OECD record. They are priorities for cognitive interviewing, translation review and content-validation studies, rather than items whose deletion or replacement can already be assumed to improve validity. Revising wording may change construct coverage, and an item with differential functioning may still represent an important facet. Any revision should be evaluated for both content representation and cross-system measurement performance.

### 4.5. Limitations

The retained structures were selected in the same systems used to evaluate them. Section 3.1 now quantifies what that costs: the selection reproduces in every random half-sample for five constructs and in 84% for imagination, but reproducibility is not adequacy, and for openness to intellect no candidate structure is adequate in any half. The reported bootstrap intervals resample systems and still do not propagate item-selection or model-selection uncertainty. Primary continuous fit statistics are computed from pairwise moments and are descriptive summaries rather than survey-robust likelihood tests, as Section 2.3 sets out; the ordinal re-estimation of Section 3.3 bounds how much that matters for all six, but its own alignment is a two-step procedure rather than a single joint model, and it addresses the complex survey design no better than the continuous analysis does. Item-matched alignment addresses item selection but is not the same as fitting the full correlated-factor structure, and separate-facet results do not validate the omitted facets or the complete constructs.

The expanded simulation increases replication precision but remains a simplified six-item, one-factor sensitivity study; it does not reproduce actual sample sizes, full factor structures or exact rotation. Planned missingness does not imply that all item nonresponse is completely at random. Student-level regressions use observed means of different item subsets and do not propagate measurement-model uncertainty. System-level models do not account for shared language, translations, region or cultural dependence, and their confidence intervals condition on estimated means. These limitations qualify the present conclusions. The two activity-participation scales and parent, teacher and school reports are outside the defined scope, so the audit does not cover the entire creativity questionnaire module.

## 5. Conclusions

The comparability of PISA 2022 creativity-related self-reports depends on the model, item set, estimator and intended inference. The continuous analyses question unidimensional scoring for four released indices and show larger scalar than metric fit decrements under the retained alternatives. Those findings do not establish metric invariance of five complete constructs under ordinal estimation. The new full-structure ordinal models do not reproduce a uniform pattern of scalar misfit, and the matched facet means differ to varying degrees across estimators. Four headline alignment estimates concern selected item subsets, and the continuous openness-to-intellect respecification remains weak. The expanded simulation is conditional evidence about a simplified design and cannot certify unbiased PISA mean comparisons. Re-estimating all six under an estimator that handles the planned missingness and the ordered response format exactly lowers every loading index and rearranges the six on it, leaves their ordering on the intercept index essentially intact, and reproduces the system means at r = 0.77 to 0.97 while moving 18% to 53% of systems by more than five rank places. Construct-level statements are reasonably robust to the estimator; the position of a particular system is not, and that asymmetry, rather than any label, is what a secondary analyst should carry away.

Within-system observed-score associations with creative-thinking performance are generally small and heterogeneous, and the joint model explains less variance than the sum of the bivariate models. These results are descriptive and do not separate measurement error, variable content mixtures and genuine moderation. Between-system correlations are additionally sensitive to ecological confounding and dependence among systems. Comparisons should therefore be presented with their measurement assumptions and sensitivity analyses, rather than licensed by a summary label. This paper no longer supplies one: the classifications it previously reported were developed after inspecting the results, and at least one of them changes side when the estimator changes.

The distinction between measurement comparability and criterion-relation comparability remains useful as a research question. The observed heterogeneity motivates latent and multilevel tests; it does not, on its own, demonstrate validity non-comparability.

## Figures and Tables

**Figure 1:**
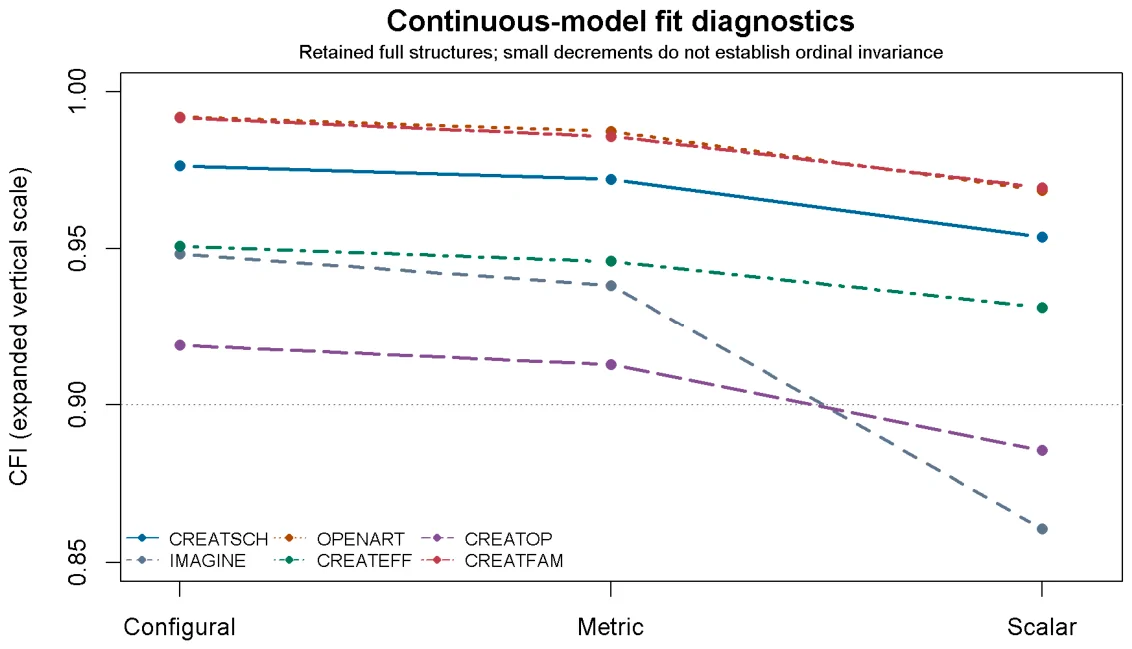
CFI for the retained continuous-variable configural, metric and scalar models. The dashed 0.90 line is a descriptive reference. Small metric decrements do not establish ordinal invariance or compensate for configural misfit; larger scalar decrements indicate departures from intercept equality within these specifications.

**Figure 2:**
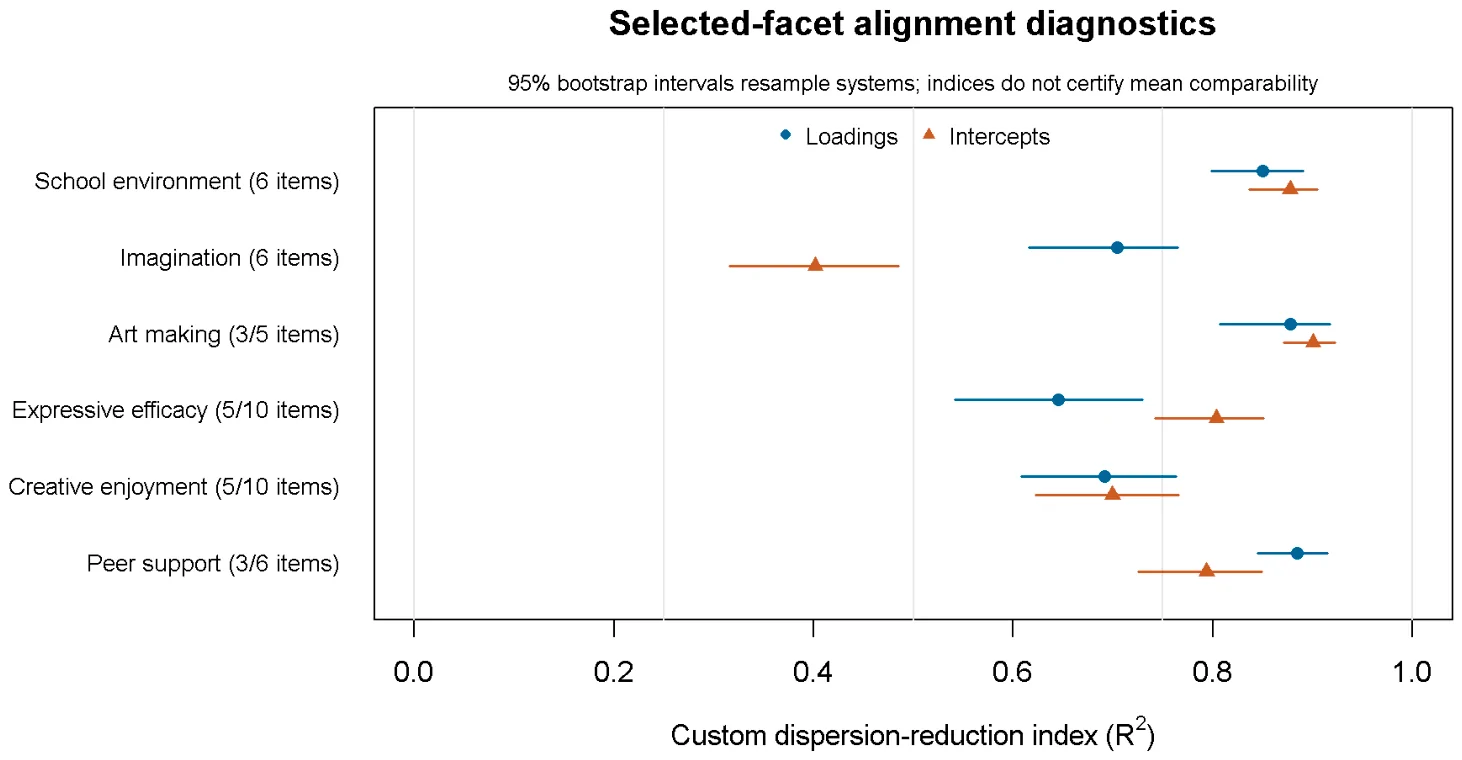
Custom alignment dispersion-reduction indices for loadings and intercepts, with 95% intervals from resampling systems. Four estimates concern selected item subsets. These indices are not direct estimates of the proportion of true construct variation or guarantees of ordinal comparability.

**Figure 3:**
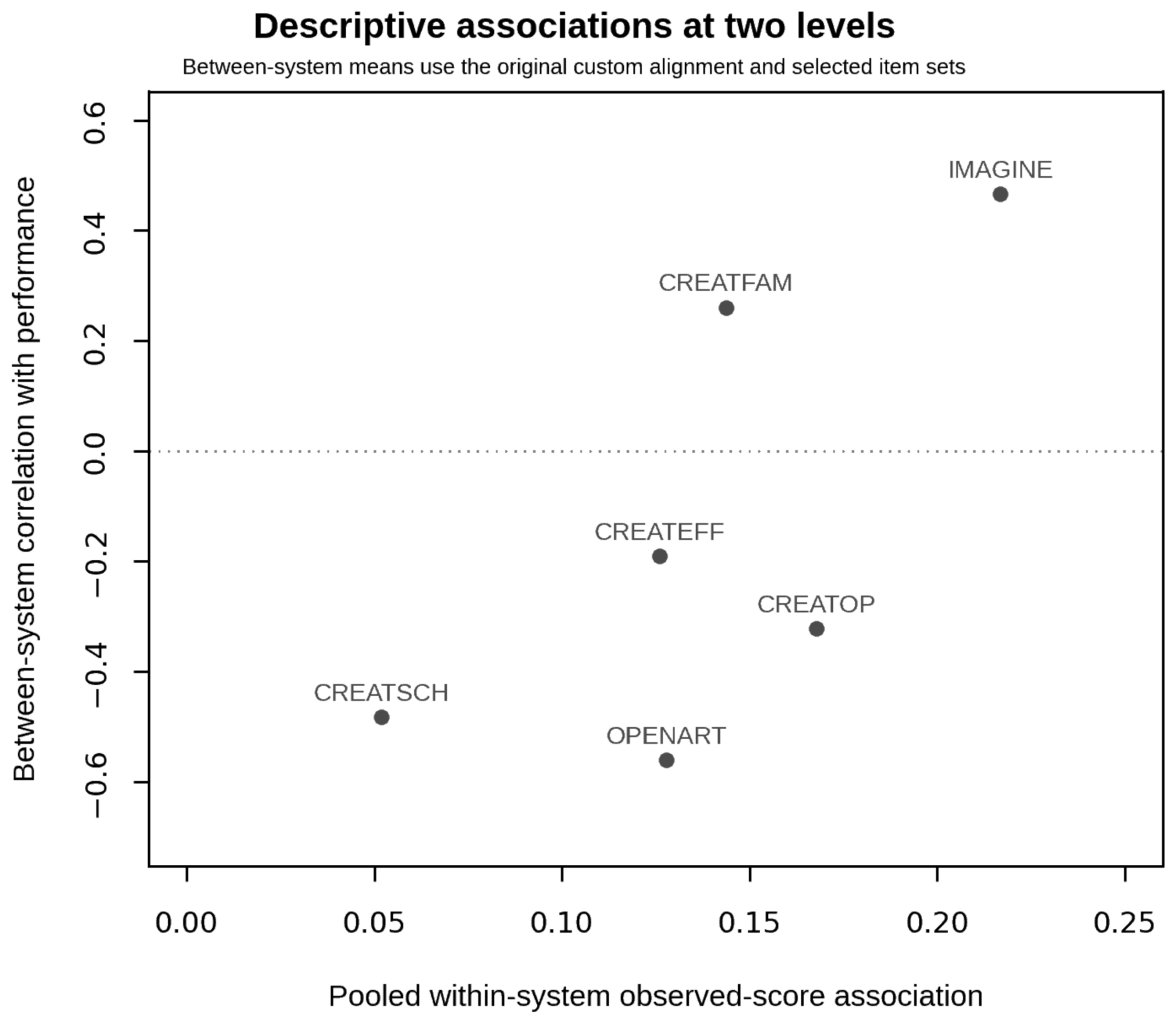
Descriptive within-system observed-score associations and between-system correlations using custom alignment means. The levels of analysis involve different estimands and different uncertainty. Between-system results depend on the selected item subsets and alignment specification, and do not account for language, regional clustering or shared translations.

**Table 1:** Analytic samples, and what the rotated design gives each student.

Construct	Items	Economies	Students	Items Answered per Student M [IQR]	Answering All Items	Scored (≥2 Items)	Implied α: Full Scale/Median Student
Creative school and class environment	6	73	468,387	4.92 [5, 5]	0.0%	99.1%	0.88/0.86
Imagination and adventurousness	6	72	454,184	4.23 [4, 5]	0.0%	99.2%	0.79/0.71
Openness to art and reflection	5	73	462,913	4.93 [5, 5]	96.5%	99.2%	0.86/0.86
Creative self-efficacy	10	72	469,651	4.93 [5, 5]	0.0%	99.1%	0.92/0.86
Creativity and openness to intellect	10	73	467,440	4.93 [5, 5]	0.0%	99.1%	0.91/0.83
Creative family and peer environment	6	72	462,943	4.93 [5, 5]	0.0%	99.1%	0.86/0.84

Note. Items = items entering our measurement models; for imagination, this is six of the seven in the published scale, the reverse-worded ST342Q01JA being excluded. Students = those answering at least one item of the scale. Items answered per student are computed over those students. Implied α applies the Spearman–Brown formula to the average inter-item correlation, computed pairwise-present because almost no student answers a whole scale, at the full item count and at the median student’s item count. Economies are those the OECD records as administering the scale (OECD, 2024b).

**Table 2:** Continuous measurement structure, fit and invariance diagnostics.

Construct	Unidimensional Fit Mdn CFI (*n* < 0.90)	Structure Retained	ω Mdn	CFI Configural [IQR Across Systems]	ΔCFI Metric	ΔCFI Scalar	Continuous Metric/Scalar
Creative school and class environment	0.976 (0 of 73)	unidimensional	0.87	0.976 [0.970, 0.982]	0.004	0.018	Small decrement/larger decrement
Imagination and adventurousness	0.953 (6 of 72)	unidimensional ‡	0.78	0.948 [0.929, 0.966]	0.010	0.078	Borderline/larger decrement
Openness to art and reflection	0.944 (0 of 73)	correlated factors	0.90	0.992 [0.989, 0.996]	0.005	0.019	Small decrement/larger decrement
Creative self-efficacy	0.888 (41 of 72)	correlated factors	0.94	0.951 [0.942, 0.962]	0.005	0.015	Small decrement/larger decrement
Creativity and openness to intellect	0.849 (57 of 73)	correlated factors §	0.92	0.919 [0.899, 0.935]	0.006	0.027	Small decrement/larger decrement
Creative family and peer environment	0.788 (68 of 72)	correlated factors	0.90	0.992 [0.989, 0.995]	0.006	0.016	Small decrement/larger decrement

Note. Unidimensional fit is the median CFI of a continuous one-factor model, with the number of systems below 0.90 in parentheses. This is not a re-estimation of the OECD IRT scaling model. For imagination, the entry concerns six analysed items; including the reverse-worded seventh item gives median CFI 0.846, with 63 of 72 systems below 0.90. Omega is McDonald’s total omega under the retained structure, summarised by its system median. Configural CFI is the pooled multi-group value, accompanied by the interquartile range of system-specific CFI values. The continuous metric decrement is compared descriptively with 0.010. Full structure-specific and system-specific results are in Supplementary Tables S5 and S6. § No candidate structure reached the 0.95 criterion for this construct in any half-sample, so the retained structure is the best admissible one rather than an adequate one. ‡ The structure selection is not reproducible: across 5000 random half-samples the unidimensional model is selected in 84% and the correlated-factor model in 16% (Section 3.1, Supplementary Table S31).

**Table 3:** Custom continuous alignment diagnostics and the new item-matched Mplus comparison.

Construct	Item Subset Aligned (Of Published Scale)	R^2^ Loadings [95% CI]	R^2^ Intercepts [95% CI]	Matched Mplus r	Non-Invariant All/Thresholds	Ordinal ML R^2^ Intercepts
Creative school and class environment	whole scale (6 of 6)	0.85 [0.80, 0.89]	0.88 [0.84, 0.91]	0.916	39.6%/48.3%	0.86
Imagination and adventurousness	analysed items (6 of 7) ‡	0.70 [0.62, 0.77]	0.40 [0.32, 0.48]	0.658	38.8%/44.1%	0.34
Openness to art and reflection	making (3 of 5)	0.88 [0.81, 0.92]	0.90 [0.87, 0.92]	0.263 †	49.0%/55.1% †	0.90
Creative self-efficacy	expressive (5 of 10)	0.64 [0.54, 0.73]	0.80 [0.74, 0.85]	0.963	32.6%/39.4%	0.73
Creativity and openness to intellect	creative (5 of 10) §	0.69 [0.61, 0.76]	0.70 [0.62, 0.77]	0.847	26.0%/32.5%	0.72
Creative family and peer environment	peers (3 of 6)	0.88 [0.85, 0.91]	0.79 [0.73, 0.85]	0.703	25.6%/33.3%	0.69

Note. R^2^ values and bootstrap intervals are dispersion-reduction indices of the continuous alignment for the item subset named in column two, not proportions of true construct variation. Matched Mplus r compares categorical with continuous Mplus means on the same items. The ordinal ML column is the full-information graded response re-estimation of Section 3.3, computed on the item-mean intercept, and covers all six constructs. † unresolved estimation warning, so that comparison is provisional. § the retained structure is the best admissible one, not an adequate one. ‡ the structure selection is not reproducible across half-samples. Tables S19, S26 and S27 report the matched comparison and the implementation audit; Tables S28–S30 the ordinal re-estimation.

**Table 4:** Expanded sensitivity simulation under a simplified six-item design.

Non-Invariant Item-by-Group Parameters	True Spread 0.15 SD (Illustrative Condition)	True Spread 0.20 SD (Illustrative Condition)	True Spread 0.24 SD (Illustrative Condition)	True Spread 0.40 SD (Illustrative Condition)
0%	r = 0.973; 27.1% misplaced; R^2^ 0.89	r = 0.984; 18.4% misplaced; R^2^ 0.93	r = 0.989; 13.1% misplaced; R^2^ 0.95	r = 0.995; 3.9% misplaced; R^2^ 0.97
25%	r = 0.934; 40.3% misplaced; R^2^ 0.38	r = 0.960; 31.0% misplaced; R^2^ 0.50	r = 0.970; 24.9% misplaced; R^2^ 0.58	r = 0.989; 11.0% misplaced; R^2^ 0.77
40%	r = 0.883; 48.0% misplaced; R^2^ 0.31	r = 0.928; 39.5% misplaced; R^2^ 0.41	r = 0.945; 34.0% misplaced; R^2^ 0.48	r = 0.979; 18.6% misplaced; R^2^ 0.70
60%	r = 0.787; 59.5% misplaced; R^2^ 0.26	r = 0.860; 52.4% misplaced; R^2^ 0.34	r = 0.895; 46.9% misplaced; R^2^ 0.40	r = 0.957; 31.0% misplaced; R^2^ 0.61

Note. 300 replications per cell; 73 groups of 1500 simulated students on six ordinal items; perturbations affect loadings and thresholds; missingness is random item masking, not the PISA rotation. r is the mean correlation of estimated with true system means; misplaced is the mean percentage of simulated systems with absolute rank error above five; R^2^ is the dispersion-reduction index. Monte Carlo standard errors and replication-level records are in Supplementary Table S17. What this design does and does not reproduce is stated in the text above.

**Table 5:** Associations of the dispositions with creative-thinking performance, within and between systems.

Construct	β Bivariate [95% CI]	τ	I^2^	SD [IQR]	β Joint [95% CI]	Systems β < 0 (Sig. Holm)	r Between Systems [95% CI]	Williams *p* (Raw/BH)	Partial r (Maths, ESCS Held)
Imagination and adventurousness	+0.217 [+0.206, +0.228]	0.040	82%	0.043 [+0.19, +0.24]	+0.159 [+0.147, +0.171]	0 (0)	+0.47 [+0.25, +0.64]	0.322/0.386	+0.40
Creativity and openness to intellect	+0.168 [+0.158, +0.178]	0.036	77%	0.044 [+0.14, +0.20]	+0.069 [+0.058, +0.080]	4 (0)	−0.32 [−0.53, −0.08]	0.002/0.005	+0.21
Creative family and peer environment	+0.144 [+0.128, +0.159]	0.060	90%	0.059 [+0.10, +0.19]	+0.078 [+0.064, +0.091]	2 (0)	+0.26 [+0.01, +0.48]	0.020/0.030	+0.02
Openness to art and reflection	+0.128 [+0.116, +0.140]	0.042	82%	0.048 [+0.09, +0.16]	−0.002 [−0.017, +0.013]	30 (6)	−0.56 [−0.71, −0.36]	0.986/0.986	−0.30
Creative self-efficacy	+0.126 [+0.114, +0.138]	0.042	82%	0.048 [+0.09, +0.17]	+0.020 [+0.007, +0.032]	25 (0)	−0.19 [−0.42, +0.06]	<0.001/<0.001	+0.25
Creative school and class environment	+0.052 [+0.031, +0.072]	0.081	94%	0.076 [−0.01, +0.10]	−0.043 [−0.059, −0.027]	50 (18)	−0.48 [−0.65, −0.27]	0.020/0.030	−0.12

Note. β bivariate and β joint are within-economy standardised associations, each estimated separately in every economy and then synthesised across economies by a DerSimonian–Laird random-effects model; the interval is the random-effects interval for the mean, τ the estimated between-system standard deviation and I^2^ the share of observed variation exceeding sampling error. SD and IQR describe the distribution of the economy-specific coefficients and replace the coefficient of variation, which is uninterpretable where the mean approaches zero. The joint model enters all six dispositions together on the 380,224 students in 62 economies with a score on every scale. Systems β < 0 counts economies with a negative joint coefficient, with the number still significant after Holm adjustment across the 62 tests in parentheses. r between systems is the economy-level correlation between mean creative-thinking performance and the original custom mean for the item set specified in Table 3, with a Fisher z interval at the realised number of systems; Williams *p* tests it against the correlation using naive means over the whole scale, raw and Benjamini–Hochberg adjusted across the six tests. The final column partials out system mean mathematics performance and system mean economic, social and cultural status. Per-economy values, the bootstrap synthesis and the item-matched version of the Williams tests are in Supplementary Tables S8 and S15.

**Table 6:** Evidence profile by construct. No categories, labels or thresholds are applied to any column.

Construct	Published Items: Mdn CFI (Systems < 0.90)	Aligned Item Subset (of Published Scale): Configural CFI	Intercept R^2^ of That Subset [95% CI]	OECD Scaling Record	Categorical–Continuous r; Criterion SD
Creative school and class environment	0.976 (0 of 73)	whole scale, 6 of 6; 0.976	0.88 [0.84, 0.91]; 0.86 under ordinal ML	no national parameters; no suppression	r = 0.916; SD 0.076
Imagination and adventurousness	0.953 (6 of 72) ‡	analysed items, 6 of 7; 0.948 ‡	0.40 [0.32, 0.48]; 0.34 under ordinal ML	7 national parameters (1.09%) in 7 groups; suppressed in 14 countries	r = 0.658; SD 0.043
Openness to art and reflection	0.944 (0 of 73)	making, 3 of 5; 0.992	0.90 [0.87, 0.92]; 0.90 under ordinal ML	16 national parameters (3.40%) in 10 groups; no suppression	r = 0.263 †; SD 0.048
Creative self-efficacy	0.888 (41 of 72)	expressive, 5 of 10; 0.951	0.80 [0.74, 0.85]; 0.73 under ordinal ML	no national parameters; no suppression	r = 0.963; SD 0.048
Creativity and openness to intellect	0.849 (57 of 73)	creative, 5 of 10; 0.919 §	0.70 [0.62, 0.77]; 0.72 under ordinal ML	20 national parameters (2.13%) in 19 groups; no suppression	r = 0.847; SD 0.044
Creative family and peer environment	0.788 (68 of 72)	peers, 3 of 6; 0.992	0.79 [0.73, 0.85]; 0.69 under ordinal ML	no national parameters; no suppression	r = 0.703; SD 0.059

Note. This table reports evidence; it does not classify. The STRONG, MODERATE, WEAK and INSUFFICIENT labels carried in earlier versions were developed after inspecting these results and have been removed rather than relabelled (Section 2.5), so no row should be read as licensing or withholding a comparison. Every figure is repeated from Table 2, Table 3 and Table 5 or from the OECD record. § no candidate structure reached the 0.95 criterion in any half-sample; the retained structure is the best admissible one, not an adequate one. ‡ the structure selection is not reproducible: the unidimensional model is selected in 84% of half-samples and the correlated-factor model in the rest. † unresolved estimation warning; that comparison is provisional. Ordinal ML values are from the full-information graded response re-estimation of Section 3.3, computed on the item-mean intercept.

## Data Availability

The student data are publicly available from the OECD PISA 2022 database (https://www.oecd.org/pisa/, accessed on 1 September 2026). The scaling tables are documented in the Technical Report Chapter 19 annexes (OECD, 2024b) (accessed on 1 September 2026), and the category-specific creative-thinking means in the published compendia (OECD, 2024a) (accessed on 1 September 2026). No new data were created in this study. The analysis code, derived tables, simulation records, Mplus inputs and parsed outputs generated for it are provided in the Supplementary Materials, which also document which inputs were run, the numerical settings, convergence and warnings, and include the data-preparation scripts and item mappings.

## Supplementary Materials

The following supporting information can be downloaded at: https://www.mdpi.com/article/10.3390/bs16101758/s1. Table S1: Item content, wording, referent classification, the OECD international item slope, and the number of country-by-language groups given a national parameter, for all 44 items of the six scales. Table S2: Item-by-group departures from the international calibration in the OECD scaling record, by scale, country-by-language group and item. Table S3: Scale reliability as reported by the OECD for each country-by-language group, for all six constructs. Table S4: Criterion gradient of every item in every system: the slope of the four published category means on the category codes, in score points per category step. Table S5: Structure comparison by construct: unidimensional, correlated-factor and bifactor specifications with median CFI and its quartiles, RMSEA, SRMR, omega, omega-hierarchical, ECV, Heywood counts, and the reason for exclusion where one applies. Table S6: Per-economy fit of each candidate structure: CFI, TLI, RMSEA, SRMR, omega, effective group size and Heywood flag. Table S7: Aligned latent means and factor standard deviations by economy, with the naive item means and the rank difference between the two. Table S8: Per-economy associations with creative-thinking performance: coefficient, total standard error, and its sampling and imputation components. Table S9: Random-effects synthesis of the per-economy associations: DerSimonian-Laird and Paule-Mandel estimates, tau, tau-squared, I-squared, Cochran Q, prediction interval, fixed-effect estimate, and a bootstrap over economies. Table S10: Item-pair co-administration counts by economy under the rotated design, with the retention decision. Table S11: Per-student coverage by construct: items answered, share scored, implied reliability at full scale and for the median student, and the share of a student’s items contributed by the headline facet. Table S12: Full-scale categorical Mplus benchmark: invariance R-squared, the number of invariant groups, and the percentage of non-invariant parameters, by construct and parameter type. Table S13: Joint model: per-economy coefficients for all six predictors entered together, with the bivariate and disattenuated coefficients and the model R-squared. Table S14: Alignment results for every eligible factor, not only the headline one: items, R-squared for loadings and intercepts with bootstrap intervals, and convergence. Table S15: Item-set-matched comparison of aligned and naive scoring, with Williams tests for the dependent correlations. Table S16: System means behind that comparison: aligned latent mean, naive mean over the whole scale, and naive mean over the aligned items only. Table S17: Expanded simulation, 300 replications per condition: mean recovery correlation, rank correlation, displacement rate and dispersion-reduction R-squared, each with its Monte Carlo standard error. The superseded 30-replication run is retained in the archive as superseded_30replication_simulation.csv. Table S18: Replication-level records behind the expanded simulation: one row per replication of each condition. Table S19: Item-matched Mplus alignment comparison: categorical against continuous means on the same items, with the percentage of non-invariant parameters and thresholds and any estimation warning. Table S20: System means from the matched comparison, by construct, estimator and economy. Table S21: Criterion correlations under the matched comparison: the original custom means against the Mplus continuous and categorical means. Table S22: Effect of excluding systems whose configural CFI falls below 0.90: intercept R-squared before and after, with the economies excluded. Table S23: Full-pattern ordinal models: configural and scalar fit, degrees of freedom, and warnings, by construct. Table S24: The same ordinal models per system: CFI, RMSEA, SRMR, warnings and printed minimum residual variance, for all 435 construct-by-system combinations. Table S25: Summary of the per-system ordinal fits: attempts, completions, and the distribution of CFI and RMSEA by construct. Table S26: Independent audit of the alignment implementation in the raw reporting metric: agreement with the Mplus solution on common configural parameters, with starts and convergence. Table S27: The same audit in the optimisation metric, with the geometric mean of the Mplus variances, the correlation, mean absolute error and largest error. Table S28: Ordinal full-information re-estimation of all six constructs beside the continuous result: R-squared for loadings and intercepts under both estimators, agreement between the system means, and the number of systems displaced by more than five ranks. Table S29: Per-system aligned means and ranks under both estimators, with the rank shift. Table S30: Cross-construct summary of what the change of estimator alters and what it leaves standing: agreement of the construct ordering on each index, the change in each, and the ordering under each estimator. Table S31: Split-system cross-validation of the structure selection over 5000 random half-samples, with the full specification curve for all three candidate structures in all six constructs. Figure S1: Distribution of Cronbach’s alpha across country-by-language groups, by construct. Figure S2: Item-by-group map of the OECD scaling record, showing where national parameters were used. Figure S3: Between-system correlations with creative-thinking performance under naive and aligned means. Figure S4: System mean criterion gradient against system mean creative-thinking performance. Every analysis script is included and runs from the supplied intermediate results. An audit folder records, for each Mplus run, whether it completed and what warning it raised, together with the superseded specifications and the software versions. The raw Mplus output files run to approximately 86 MB across 24 runs and are in the repository deposit rather than the archive; the inputs, item keys and parsed results are in the archive, and the supplied parser regenerates every reported number from the output. Student-level data are the public-use PISA files, and the preparation scripts that derive the Mplus data from them are included.
